# An Unusual Case of Serotonin Syndrome with Oxycodone and Citalopram

**DOI:** 10.1155/2012/261787

**Published:** 2012-05-29

**Authors:** Clare Walter, David Ball, Mary Duffy, James D. Mellor

**Affiliations:** ^1^Pharmacy Department, Peter MacCallum Cancer Centre, St. Andrew's Place, East Melbourne, VIC 3002, Australia; ^2^Division of Radiation Oncology and Cancer Imaging, Peter MacCallum Cancer Centre, St. Andrew's Place, East Melbourne, VIC 3002, Australia; ^3^Division of Cancer Nursing, Peter MacCallum Cancer Centre, St. Andrew's Place, East Melbourne, VIC 3002, Australia

## Abstract

A 77-year-old female with recurrent non-small-cell lung cancer presented to a hospital outpatient clinic with tremor, weakness, inability to coordinate motor movements, and confusion. It was suspected that the symptoms were due to possible central nervous system metastases; however, a CT scan of her head was unremarkable. The lung clinic liaison pharmacist took a medication history from the patient, complimented by extra information from the patient's community pharmacy. The pharmacist suspected the rare side effect of serotonin syndrome was responsible for the patient's presenting symptoms caused by the combination of oxycodone and citalopram. The patient's symptoms resolved soon after oxycodone was changed to morphine.

## 1. Introduction

Oxycodone is an opioid analgesic widely used in the treatment of moderate to severe cancer-related pain. It is available in immediate- and sustained-release oral formulations. Depression is also a disabling comorbidity which is common in patients with malignancies, particularly those who have advanced or metastatic disease. It is estimated that up to 15–25% of cancer patients suffer from depression [1]. Citalopram is a selective serotonin reuptake inhibitor licensed to treat the symptoms of depression.

## 2. Case Presentation

A 77-year-old female with recurrent non-small-cell lung cancer presented to the outpatient lung clinic complaining of tremor, weakness, inability to coordinate motor movements, and confusion. Central nervous system metastases were suspected, and a CT scan of the head was ordered.

The patient's medications were reported as oxycodone (slow release) 50 mg twice daily, oxycodone (immediate release) 5 mg when required, esomeprazole 40 mg twice daily, temazepam 10 mg at night, and docusate 100 mg with sennosides 16 mg twice daily. There was confusion as to whether this list was complete.

A telephone conversation with the patient's community pharmacist revealed in addition to her reported medications that she had recently picked up a repeat prescription of citalopram 20 mg once a day, after not having it dispensed for several months. Diazepam had also recently been prescribed by her general practitioner for restless legs. The oxycodone had been started several months earlier (rotated from morphine) for cancer-related pain during the period that the patient was not taking her citalopram.

Further discussion with the patient elucidated that the symptoms started shortly after recommencing the citalopram. The lung clinic pharmacist suspected a drug interaction between citalopram and oxycodone which had resulted in serotonin syndrome. Use of the Naranjo probability scale indicated a probable relationship between the combination of oxycodone and citalopram and the serotonin symptoms [2]. The symptoms satisfied the Sternbach diagnostic criteria for serotonin syndrome [3].

Oxycodone was changed back to morphine, and the esomeprazole was reduced to 40 mg daily (which in turn should improve citalopram clearance). The symptoms resolved within 48 hours, and the CT scan later came back clear.

## 3. Discussion

Serotonin syndrome is a serious adverse reaction thought to result from hyperstimulation of brainstem 5-HT1A and 2A receptors [4]. Coadministration of oxycodone with serotonin reuptake inhibitors has only recently been associated with the development of serotonin syndrome. An extensive literature search revealed only four previous reports of serotonin syndrome involving oxycodone [5, 6]. The opioids of the phenylpiperidine series: pethidine, tramadol, methadone, fentanyl, dextromethorphan, and propoxyphene are weak serotonin reuptake inhibitors and are thus known to have an association with serotonin symptoms [7]. Oxycodone is a morphine analogue and so does not fall into this category of opioids. An alternative explanation is required.

Oxycodone differs from most other opioids in having a significant affinity for kappa opioid receptors [8]. While most of the drug is N-demethylated by CYP 3A4 to noroxycodone, approximately 10% is O-demethylated by CYP 2D6 to the very potent active metabolite, oxymorphone [8, 9]. Compared to oxycodone, oxymorphone has a higher affinity for the mu opioid receptor and is 14 times as potent as oxycodone [10].

Other potential contributing factors for the drug interaction include the following:

- a pharmacokinetic interaction between citalopram and esomeprazole, reducing the citalopram clearance. However, the patient had been on this combination for sometime without experiencing serotonin effects [11–13];
- the inhibition of CYP 2D6 by citalopram affecting the metabolism of oxycodone. However, this inhibition is only classified as “weak” [9]. Available data indicates that impaired activity of CYP 2D6 is associated with only small changes in oxycodone pharmacokinetics, which do not usually result in altered oxycodone pharmacodynamics [14]. Also, if there were any effect, it would result in less of the active oxymorphone metabolite, rather than more;
- opioids do not directly stimulate serotonergic neuronal discharge but disinhibit neuronal activity by suppressing GABA-mediated inhibition [15]. Thus, the short-term effect of morphine and perhaps other opioids is to increase serotonin release in widespread areas of the forebrain [16]. However, the patient had been on the same dose of oxycodone for 3 months prior to the onset of symptoms;
- the patient may have been an extensive CYP 2D6 metaboliser. Whilst the metabolism of oxycodone to oxymorphone via CYP 2D6 is not responsible for all of the effects of oxycodone, experimental results indicate it does make a significant contribution in extensive CYP 2D6 metabolisers [17].

Table 1 summarises the activity of the relevant isozymes of the hepatic cytochrome P450 system [9, 12–14].

## 4. Conclusion

Whilst serotonin syndrome is not listed as an adverse event within the product information for OxyContin tablets when given in combination with a selective serotonin reuptake inhibitor, the small number of existing case studies to date suggests that it is prudent to closely monitor patients receiving this combination. The mechanism of interaction is unclear and warrants further investigation.

## Figures and Tables

**Table 1:** 

Drug	Metabolised by CYP	Inhibits CYP
Citalopram	3A4 2C19	1A2, 2D6, 2C19 (weak)
Esomeprazole	2C19, 3A4	2C19
Oxycodone	3A4 (to form noroxycodone) 2D6 (to form oxymorphone)	
